# Tailoring Co Distribution in PtCo Alloys for Enhanced Oxygen Reduction Reaction Activity and Durability in Fuel Cells

**DOI:** 10.3390/nano15090657

**Published:** 2025-04-26

**Authors:** Jinhee Lee, Miso Kim, Bongho Lee, Jeonghee Jang, Suhwan Lee, Dae Jong You, Juseok Song, Namgee Jung

**Affiliations:** 1 Carbon Inc., 4, Techno 2-ro, Yoseong-gu, Daejeon 34015, Republic of Korea; ljh001@thecarbon.studio (J.L.); misokim@thecarbon.studio (M.K.); leebh@thecarbon.studio (B.L.); chemswatt@thecarbon.studio (J.J.); limm741@thecarbon.studio (S.L.); daejong73@thecarbon.studio (D.J.Y.); juseok@thecarbon.studio (J.S.); 2 Graduate School of Energy Science and Technology (GEST), Chungnam National University, 99 Daehak-ro, Yuseong-gu, Daejeon 34134, Republic of Korea

**Keywords:** polymer electrolyte membrane fuel cell, PtCo alloy, colloidal Pt nanoparticles, near-surface structure

## Abstract

In polymer electrolyte membrane fuel cells (PEMFCs), substantial efforts have been made to focus on Pt and Pt alloy catalysts to enhance their catalytic performance. However, these catalysts still fail to meet practical requirements and existing PtCo catalysts face durability issues due to structural limitations. In this study, carbon-supported hybrid PtCo alloy catalysts (H-PtCo) with improved activity and durability are synthesized by reducing Co precursors onto pre-formed colloidal Pt nanoparticles. Elemental mapping via transmission electron microscopy reveals that the H-PtCo catalysts exhibit a high concentration of Co atoms near the sub-surface. This Co enrichment results from the conformal deposition of Co atoms onto Pt nanoparticles, followed by high-temperature treatment. Electrochemical characterizations, including linear sweep voltammetry (LSV) and accelerated durability test (ADT), demonstrate that the H-PtCo catalysts outperform conventional PtCo alloys (C-PtCo), synthesized via the co-reduction method of Pt and Co, in terms of oxygen reduction reaction (ORR) activity and stability. Furthermore, single-cell tests reveal that the H-PtCo catalysts significantly enhance both performance and durability compared to C-PtCo and Pt catalysts. These findings emphasize the critical role of Co atom distribution within PtCo nanoparticles in improving catalytic efficiency and long-term stability.

## 1. Introduction

As the importance of carbon dioxide reduction grows increasingly urgent, the adoption of renewable energy technologies, particularly fuel cells, is expanding. Among the various fuel cell technologies, polymer electrolyte membrane fuel cells (PEMFCs) are of significant interest due to their high efficiency, low operating temperatures, and versatility across diverse applications, including transportation, power generation, and building energy systems [1].

In PEMFCs, Pt and its alloys have long been recognized as the most effective electrocatalysts for the oxygen reduction reaction (ORR), owing to their superior catalytic activity and stability. However, the high cost and limited availability of Pt remain major obstacles to the widespread commercialization of PEMFCs. In response, considerable efforts have been directed towards developing alternative catalysts that can deliver comparable ORR performance with reduced reliance on Pt, thus addressing both cost and resource constraints [2,3,4].

A widely employed strategy to enhance ORR activity of Pt is to modify its electronic structure through ligand and strain effects induced by alloying transition metals such as Fe, Co, Ni, Cu, and others [5,6,7]. This modification alters the oxygen binding energy, resulting in significantly improved activity compared to bare Pt [8,9,10]. However, transition metals in these alloy catalysts are prone to electrochemical leaching during fuel cell operation due to rapid oxidation, leading to performance degradation [11,12,13].

According to previous studies, dealloying has been proposed as an effective strategy to mitigate the leaching of transition metals. Dealloying refers to the selective dissolution of specific metals from an alloy, thereby removing surface impurities or undesirable metallic constituents. Through this process, 3d transition metals, prone to dissolution, can be effectively removed from the particle surface, leading to significantly enhanced catalyst stability. One of the key advantages of dealloying is that the catalyst surface structure can be modified while preserving its catalytic performance. This approach is particularly effective for addressing issues associated with Co leaching in Pt-Co alloys. By selectively removing Co, Pt can be retained in a relatively stable state, which in turn enhances the durability of the catalyst toward ORR [14].

However, during the dealloying process, the non-uniform removal of constituent elements from the catalyst surface can lead to increased surface roughness. For instance, during the acid-induced dealloying of Pt-based alloy nanoparticles with non-uniform atomic arrangements, it can be challenging to precisely modulate the near-surface concentration of 3d transition metals and to tailor the structural characteristics of the resulting Pt-enriched surface. Such morphological changes compromise the structural stability of the catalyst under electrochemical conditions and may induce particle rearrangement or agglomeration during the long-term operation of PEMFCs. As a result, surface structural degradation may occur, leading to the loss of active sites and hindering electrode reactions, ultimately causing a decline in overall catalyst durability. Therefore, to fundamentally overcome these challenges, it is imperative to develop innovative synthetic strategies that can concurrently ensure structural stability and high catalytic performance [15].

A commonly employed technique for synthesizing Pt-based alloy catalysts is the polyol method, which offers several advantages, including simplicity, scalability, and the ability to control particle size and morphology [16,17,18,19]. In this approach, Pt and transition metals such as Co are typically reduced simultaneously in a polyol solution, leading to the formation of PtCo alloy nanoparticles. However, the co-reduction process often results in insufficient control over the distribution of alloying components, causing Co aggregation at the surface or its leaching during fuel cell operation [20,21,22]. These structural inhomogeneities significantly compromise the catalytic activity and long-term stability of the catalysts [22].

**Figure 1:**
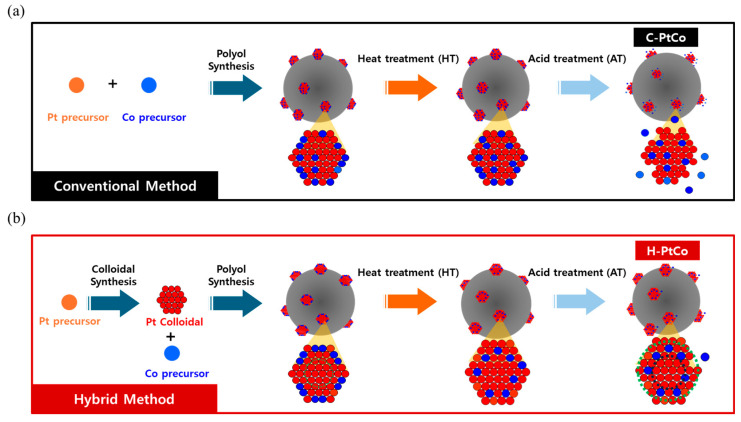
Schematic illustration of (**a**) the conventional method for synthesizing C-PtCo catalysts and (**b**) the hybrid method for preparing H-PtCo catalysts.

In this study, to overcome the limitations inherent in the co-reduction approach, we propose an alternative synthesis strategy involving the formation of Pt colloidal nanoparticles followed by the sequential reduction in Co and high-temperature treatment (Figure 1). This method allows for more precise control over the distribution of Co atoms within the Pt matrix, particularly enriching the near-surface region with Co atoms. The Co-enriched sub-surface optimizes the electronic structure and lattice strain effects, thereby promoting enhanced ORR kinetics. By minimizing direct exposure of Co on the surface, this design strategy effectively can mitigate Co dissolution, further contributing to the long-term robustness of the catalyst. The resulting catalysts exhibit enhanced ORR activity and stability, as the controlled Co distribution minimizes the tendency for Co segregation and leaching, which are common drawbacks in co-reduced PtCo catalysts.

## 2. Materials and Methods

### 2.1. Preparation of C-PtCo via Conventional Polyol Method

A total of 1.95 g of carbon black (Li-435, Denka black Inc., Tokyo, Japen) was added to 1056 g of ethylene glycol (98% ethylene glycol, Sigma Aldrich, Louis, MO, USA) solution, and the carbon dispersion solution was obtained by ultrasonic/high-speed dispersion. To the carbon dispersion solution was added 16.24 g of Pt precursor (10% (MEA)₂Pt(OH)₆ TNI Chem Co., Ulsan, Republic of Korea), 1 g of dispersant (50% sodium hypophosphite monohydrate, Sigma Aldrich., USA), and 97 g of cobalt precursor (1% cobalt chloride, Sigma Aldrich, Louis, MO, USA) solution were placed in an autoclave reactor equipped with a stirrer and the reduction reaction was carried out for 4 h. Upon completion of the reaction, the slurry was filtered and washed several times with deionized water (D.I. water) and then subjected to heat treatment at 600 °C under H_2_ gas atmosphere for 2 h and subsequently at 650 °C under N_2_ atmosphere for 1 h. After heat treatment, the slurry was subjected to acid treatment in 1 M HNO₃ solution at 90 °C for 1 h. Afterwards, filtration and washing were repeated with excess D.I. water, followed by freeze drying at −35 °C. Finally, the C-PtCo powder was obtained by sieving through a sieve size of 100 μm.

### 2.2. Preparation of H-PtCo via Polyol-Based Hybrid Method

A total of 16.23 g of platinum precursor (10% (MEA)₂Pt(OH)₆, TNI Chem Co., Ulsan, Republic of Korea), 1 g of dispersant (50% sodium hypophosphite monohydrate, Sigma Aldrich, USA) and 1000 g of ethylene glycol (98% ethylene glycol, Sigma Aldrich, Louis, MO, USA) were placed in a reflux type glass reactor and heated at 110 °C for 1 h to prepare the platinum colloidal form. Then, 1.95 g of carbon black (Li-435, Denka Black Inc., Tokyo, Japen) and 24.38 g of cobalt precursor (4% cobalt chloride, Sigma Aldrich, Louis, MO, USA) solution were mixed in this solution, and ultrasonic/high-speed dispersion was performed for 20 min to prepare a carbon dispersion solution. The resulting dispersion solution was placed in an autoclave reactor equipped with a stirrer, and the temperature of the reactor was heated to about 250 °C to conduct the reduction reaction. After the reaction was completed, the slurry was filtered and washed repeatedly with copious D.I. water, and then heat-treated in a furnace at 600 °C under H_2_ gas atmosphere for 2 h and subsequently at 650 °C under N_2_ atmosphere for 1 h. The slurry was then stirred in a 1 M HNO₃ solution for acid treatment at 90 °C for 1 h, filtered and washed with excess D.I. water. Finally, freeze-drying was performed and the H-PtCo powder was obtained by sieving through a sieve size of 100 μm.

### 2.3. Physical Characterization

Various analytical instruments were utilized to characterize the physicochemical properties of the C-PtCo and H-PtCo catalysts. The size and distribution of catalyst particles were confirmed using a transmission electron microscope (TEM, JEM-2100F HR, 200 kV, Jeol Ltd., Tokyo, Japan), and the elemental mapping within the catalyst particles was conducted using Cs-TEM (Titan Cubed G2 60-300, FEI Company, Hillsboro, OR, USA) and energy dispersive X-ray spectroscopy (EDS) for elemental distribution analysis. In addition, X-ray diffraction (XRD, SmartLab SE, Rigaku Co., Tokyo, Japan) was used to analyze the crystal structure and alloy formation degree of each catalyst, and X-ray photoelectron spectroscopy (XPS, K-alpha+, Thermo Fischer Scientific, Waltham, MA, USA) was used to confirm the chemical composition and electronic structure of the catalyst surface.

### 2.4. Half-Cell Test

The electrochemical measurements were carried out using a three-electrode system consisting of an Ag/AgCl reference electrode, a rotating disk electrode (RDE) with a glassy carbon electrode (working electrode), and a Pt wire (counter electrode). The catalytic ink slurry was prepared by mixing 5 mg catalyst, 70 μL Nafion ionomer (5 wt%, Sigma-Aldrich), and 900 μL 2-propanol (99.5%, Sigma-Aldrich) and dispersed by ultrasonic dispersion. From the prepared ink, 5 μL was loaded onto the glassy carbon electrode (0.196 cm^2^, geometric surface area) and allowed to dry. The slurry-coated RDE was then connected to the device, and the cyclic voltammograms (CVs) and the ORR polarization curves were measured while immersed in 0.1 M HClO₄ electrolyte. For the CVs, N_2_ gas was purged for 20 min, and the change in currents depending on the applied potential was recorded in the range of 0.05–1.05 V_RHE_ at a 20 mV s^−1^ scan rate while injecting ultra-pure N_2_ gas into the 0.1 M HClO₄ solution. The electrochemically active surface area (ECSA) was calculated by analyzing the hydrogen adsorption/desorption region in the CVs using the following equation [23]:

$$ECSA\left(m^{2}\;g_{Pt}^{-1}\right)=\frac{Q_{H}(mC)}{0.21mC\;{\mathrm{cm}}^{-2}\times L_{Pt}(mg)}$$

where Q_H_ represents the charge associated with hydrogen adsorption/desorption, which is measured through CV. The value of 0.21 mC cm⁻^2^ is the theoretical charge required for hydrogen adsorption/desorption per unit area of Pt. L_Pt_ denotes the total mass of Pt loaded on the electrode. ORR measurements were performed at 5 mV s^−1^ scan rate with a rotational speed of 1600 rpm in O_2_-saturated 0.1 M HClO₄ solution, while the potential range was kept the same. In addition, accelerated durability tests (ADTs) were performed at 100 mV s^−1^ scan rate in N_2_-saturated 0.1 M HClO₄ solution, with 10,000 CV cycles in the range of 0.6–1.0 V_RHE_. After the ADT, the ORR activity of each sample was evaluated once again and compared to that of the fresh sample to assess durability.

### 2.5. Single-Cell Test

For the single-cell performance tests, two membrane electrode assemblies (MEAs) were fabricated using C-PtCo and H-PtCo samples as the cathode catalysts, respectively, while commercially available Pt/C was applied as the anode catalysts in both MEAs. The catalyst slurry was prepared as follows: In a Jar Mill, 0.5 g of catalyst, 2.4 g of 11 wt% Nafion ionomer solution (Sigma-Aldrich), 2.2 g each of DI water and dipropylene glycol (98%, Sigma-Aldrich) were added, with the I/C ratio (ionomer/carbon) set to 1. The mixture was then ball milled at a speed of 250 rpm for 3 h to uniformly disperse the catalyst, and the catalyst slurry was coated in a 25 cm^2^ area. The alloy catalysts of 0.2 mg cm^−2^ were loaded on the cathode, whereas 0.05 mg cm^−2^ of Pt/C catalyst was coated on the anode. The electrodes were dried at 60 °C in an oven for 8 h, and then the catalyst-coated electrodes and membrane (Nafion 211, Chemours, Wilmington, Delaware USA) were hot-pressed at 165 °C and 20 bar using a vacuum press to prepare a catalyst-coated membrane (CCM). The single-cell performance was evaluated under the following conditions: stoichiometric ratio (SR) of H_2_ = 1.5, 40% RH for the fuel electrode, SR of air = 2.0, 40% RH for the air electrode, cell temperature = 80 °C, and 150 kPa pressure. The durability tests were then carried out up to 30,000 cycles in a H_2_/N_2_ atmosphere at a cell temperature of 80 °C, 100% RH, and ambient pressure, with 1 cycle at 0.6 V for 3 s and 3 s at 0.95 V for 3 s. After the durability tests, the polarization curves of the single cells were re-measured under the same conditions as the previous performance tests.

## 3. Results and Discussion

As illustrated in Figure 1a, the C-PtCo catalyst is synthesized Via the simultaneous reduction in Pt and Co precursors, leading to the formation of a randomly distributed alloy structure. During subsequent thermal and acid treatment, the dissolution of unstable surface Co atoms occurs, resulting in an increase in surface roughness. This structural characteristic may significantly influence the electrocatalytic performance and long-term durability of the catalyst. In contrast, the H-PtCo catalyst, depicted in Figure 1b, is synthesized through a sequential synthesis approach. Initially, the Pt precursors are reduced to generate colloidal Pt nanoparticles, followed by the introduction of Co precursors, which selectively deposit onto the Pt surface. Through post-synthetic thermal annealing, Co atoms diffuse into the Pt lattice, facilitating the sub-surface migration of Co from the outermost layer. A subsequent acid leaching step selectively removes residual surface Co, yielding a Pt-enriched shell with a Co-rich sub-surface structure. This distinct structural configuration is expected to endow the H-PtCo catalyst with superior electrochemical activity and durability.

**Figure 2:**
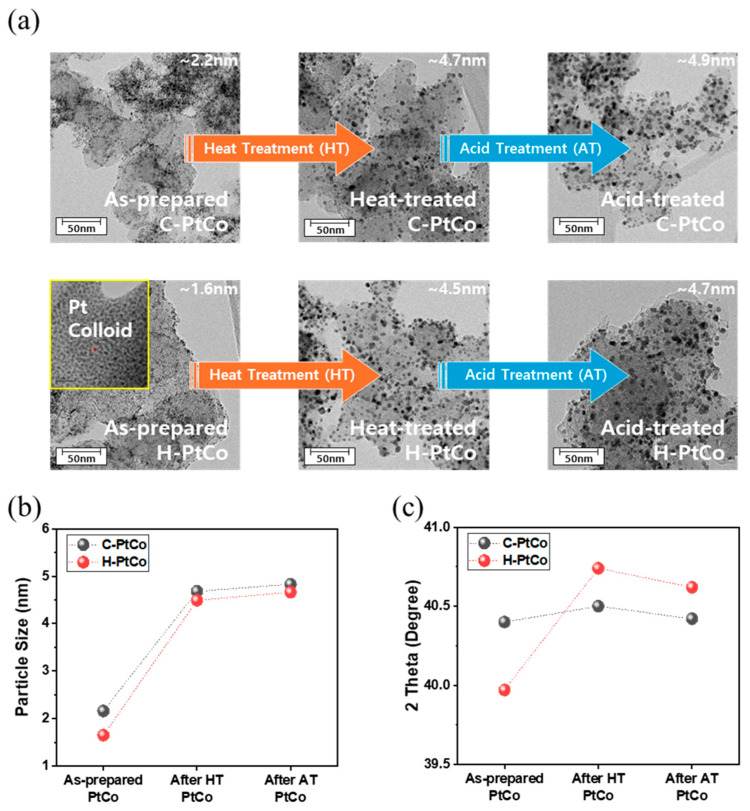
(**a**) TEM images of the C-PtCo and H-PtCo catalysts captured in sequential fabrication steps: as-prepared, heat treatment, and acid treatment. (**b**) Change in the particle size of the samples in the corresponding fabrication steps based on the TEM images. (**c**) Change in the 2θ values for the Pt(111) facet of each catalyst in the corresponding fabrication steps.

Figure 2a presents TEM images illustrating the changes in the average particle size and particle dispersion observed during the subsequent synthesis process of C-PtCo and H-PtCo catalysts. Immediately after synthesis, it can be seen that small nanoparticles (1.5–2.5 nm) were uniformly dispersed on the carbon supports. After heat treatment at 600 °C, each catalyst showed a slightly increased particle size of ~4.5 nm, while after acid treatment, the particle size and distribution of the catalysts were hardly changed as compared to the heat-treated samples (Figure 2b). The average particle size and particle distribution from the TEM images shown in Figure 1 are presented as graphs for each synthesis process (Figure S1).

Furthermore, the crystal structures of each catalyst were analyzed by XRD and the results are shown in Figure 2c. To clearly illustrate the structural changes throughout the synthesis process, the variations in 2θ values at each stage were visualized based on the XRD patterns of the catalysts (Figure S3). The analysis showed that the 2θ values for the Pt(111) facet of the as-prepared C-PtCo and H-PtCo catalysts differed by 40.4 and 39.97, respectively, which is due to the difference in the catalyst synthesis method: in the case of the C-PtCo catalyst, Pt and Co are reduced simultaneously, and the inclusion of Co into the Pt lattice causes the shrinkage of the Pt lattice, resulting in a positive shift in the 2θ value from 39.8 (a bare Pt) to 40.4 [24,25]. In contrast, for the H-PtCo catalyst, since Pt colloidal particles are formed first and then Co is reduced on the Pt nanoparticles supported by carbons, Co atoms are mainly deposited on the surface of Pt and are partially alloyed with Pt nanoparticles, exhibiting relatively smaller 2θ values in the XRD patterns compared to the C-PtCo catalyst.

For the catalysts subjected to high-temperature heat treatment, we found that the degree of alloying is more enhanced, especially in the H-PtCo catalyst compared to the C-PtCo catalyst. The C-PtCo catalyst is expected to have disorderly distributed Co atoms on both the metal nanoparticles and the carbon supports since Pt and Co with different reduction potentials undergo random co-reduction [26,27,28]. However, it is believed that for the H-PtCo catalyst, Co can be well alloyed with Pt nanoparticles during heat treatment since most of Co atoms are directly deposited on the pre-formed Pt nanoparticles during the second-step synthesis. Finally, after the acid treatment step, the degree of alloying of each catalyst tends to decrease due to the elution of a trace amount of unstable Co atoms from the surface, decreasing the 2θ values in their XRD patterns.

**Figure 3:**
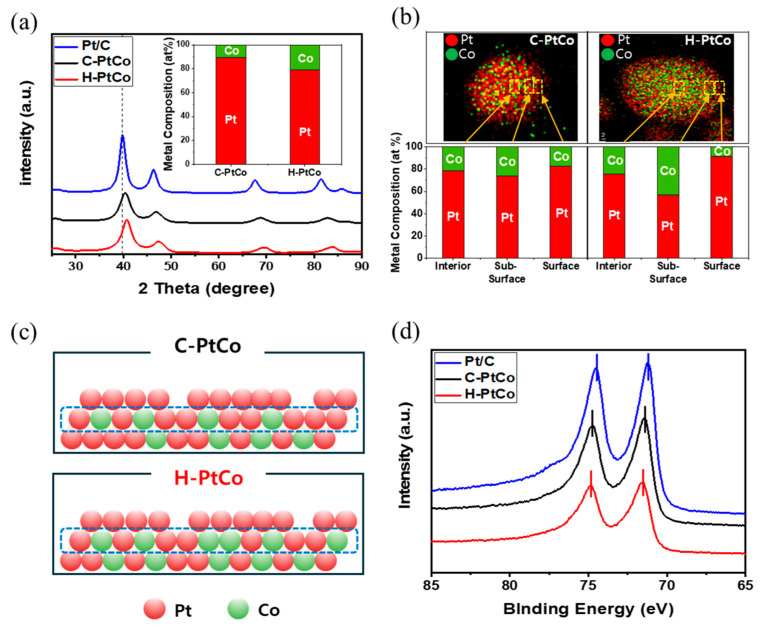
(**a**) XRD patterns (the inset indicates the bulk metal composition calculated from the 2θ values for the Pt(111) facet in the XRD patterns using Vegard’s law), (**b**) EDS mapping images and space-resolved elemental composition obtained from the corresponding EDS spectra, (**c**) the suggested surface structure model, and (**d**) Pt4f XPS spectra of the H-PtCo and C-PtCo catalysts.

Figure 3a shows the XRD patterns of the C-PtCo and H-PtCo catalysts finally obtained after heat and acid treatments. The Pt/C catalyst exhibits a Pt(111) peak at 39.8°, while the Pt(111) peaks of both the alloy catalysts are positively shifted. This is consistent with the trend typically reported in the literature for Pt-based alloy catalysts, as previously discussed in Figure 2c [24,25,27,28]. In particular, the H-PtCo catalyst shows a further shifted Pt(111) peak to the right than the C-PtCo catalyst, indicating that the H-PtCo catalyst is alloyed with relatively more Co than the C-PtCo catalyst. To quantitatively determine the ratio of Pt to Co, Bragg’s law was used to calculate the respective lattice constants, which were 3.88 Å for the C-PtCo catalyst and 3.84 Å for the H-PtCo catalyst [29]. In addition, Vegard’s law was applied to estimate the metal ratio in the alloy, which was calculated to be Pt:Co = 89:11 for C-PtCo and Pt:Co = 79:21 for H-PtCo [30]. It can be seen that in the case of the C-PtCo catalyst, Pt and Co are randomly distributed, facilitating faster elution of Co atom during acid treatment, whereas the H-PtCo catalyst has well-structured Pt-Co bonds inside the nanoparticles and especially has abundant Co atoms in the sub-surface of the Pt shell, suppressing the elution of Co atoms during acid treatment (the inset of Figure 3a).

The comparison of Co atom distribution in Pt alloy catalyst was investigated by TEM-EDS for H-PtCo/C and C-PtCo/C catalysts, as shown in Figure 3b. It is observed that the C-PtCo catalyst has a uniform distribution of cobalt atoms, but the H-PtCo catalyst has a richness of Co atom in the sub-surface of Pt, which is attributed to the unique hybrid synthesis process that reduces additional Co precursors to the pre-formed colloidal Pt nanoparticle. Those structures could form a more favorable compressive strain on Pt to the contraction by the alloy of Pt and Co. The apparently positive effect of the H-PtCo structure enables to tune the geometric properties of Pt in the alloy catalyst.

Using the EDS, we determined the elemental composition of the alloy nanoparticles at different position interior, sub-surface layers, and outmost surface layers, as shown in Figure 3b. Indeed, the H-PtCo catalyst clearly exhibits much more Co atoms detected at the sub-surface layers compared to the C-PtCo catalyst. Furthermore, a trace amount of Co atoms is expected to be dissolved only from the outmost surface during the acid treatment, leading to the formation of the Pt shell-like surface structure. Based on these findings, we propose a well-designed surface structure with Pt shell and Co-enriched sub-surface layers for the H-PtCo catalyst, as shown in Figure 3c. In contrast, the C-PtCo catalyst prepared by a conventional co-reduction method might retain less amount of Co atoms in the sub-surface layers due to severe dissolution during acid treatment.

Unambiguously, the XPS spectra of the samples (Figure 3d) exhibit a pronounced alteration in the electronic structure of Pt within the PtCo alloy catalysts. As reported in a previous study [31], the most intense doublet peak of Pt 4f7/2 for P/C, observed at 71.1 eV, can be ascribed to metallic Pt. However, the XPS spectra of both the C-PtCo and H-PtCo catalysts show a shift towards higher binding energies compared to Pt/C. Considering that the XPS spectra primarily capture the electronic environment of the surface and near-surface regions, the degree of this shift provides insight into the spatial distribution of Co within the catalyst matrix [32,33]. Notably, the H-PtCo catalyst exhibits a more pronounced increase in binding energy compared to the C-PtCo catalyst, suggesting a preferential localization of Co atoms near the Pt surface, particularly within the sub-surface region. In addition, oxidized Pt species such as Pt^2^⁺ and Pt^4^⁺ typically located at higher binding energies than metallic Pt^0^ also exhibited a similar shift toward higher energies (Figure S4). This consistent trend across all oxidation states of Pt further supports the notion that alloying with Co induces a systematic modification of the electronic structure of Pt throughout the near-surface region of the catalyst [34]. This structural modification is expected to play a pivotal role in modulating the catalytic activity and durability of PtCo alloy catalysts.

**Figure 4:**
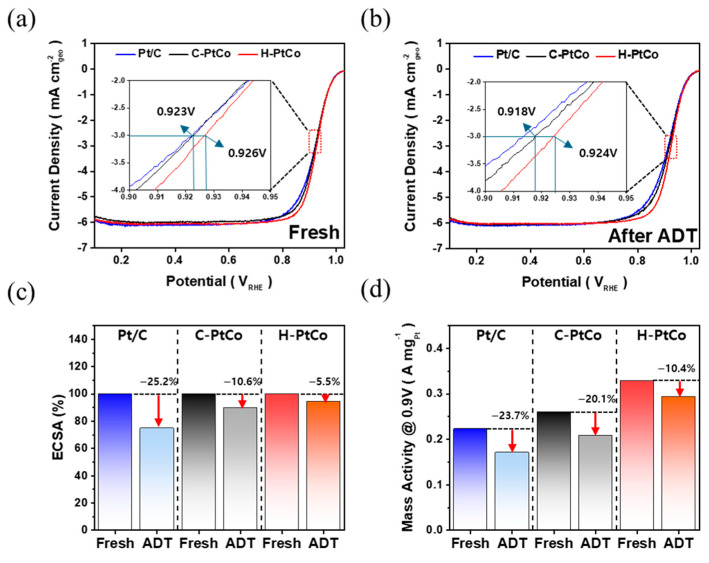
ORR polarization curves of Pt/C, C-PtCo, and H-PtCo catalysts (**a**) before and (**b**) after ADTs. (**c**) Change in ECSA of the catalysts during ADTs. (**d**) Change in mass activity of the catalysts during ADTs.

To investigate the relationship between the distribution of Co atoms within PtCo catalysts and the ORR activity, the polarization curves of Pt/C, H-PtCo, and C-PtCo catalysts were measured in O₂-saturated 0.1 M HClO₄ solution (Figure 4a). The results show that the half-wave potential (E_1/2_) of the C-PtCo catalyst is 0.923 V, while that of the H-PtCo catalyst shows a higher E_1/2_ of 0.926 V (the inset of Figure 4a). Furthermore, a comparison of the Tafel slopes for C-PtCo (83.9 mV dec⁻^1^) and H-PtCo (75.2 mV dec⁻^1^) shows that the H-PtCo catalyst exhibits a lower slope (Figure S5), suggesting superior ORR activity and charge transfer efficiency compared to the C-PtCo catalyst [35]. Therefore, the higher activity of the H-PtCo catalyst can be elucidated by the modification of the Pt electronic structure due to lattice contraction induced by the abundant Co atoms in the sub-surface layers, as suggested in Figure 3.

Additionally, as shown in Figure 4b, ADTs were performed by applying 10,000 CV cycles in the potential range of 0.6 to 1.0 V at a scan rate of 50 mV s⁻^1^ in N₂-saturated 0.1 M HClO₄ solution. As shown in the inset of Figure 4b, the E_1/2_ of the C-PtCo catalyst significantly decreased by 5 mV from 0.923 to 0.918 V during the ADT, while that of the H-PtCo catalyst slightly shifted by 2 mV from 0.926 to 0.924 V. These results suggest that the near-surface structure of the PtCo catalyst contributes to maintaining high activity even during long-term operation. As a result, the H-PtCo catalyst with Pt shell-like surface and a Co-rich sub-surface layer demonstrates improved stability, which can be elucidated by slower Co dissolution at the surface compared to the C-PtCo catalyst with a random alloy structure. Meanwhile, as shown in Figure 4c, the ECSAs of the Pt/C, C-PtCo, and H-PtCo catalysts decrease by 25.2, 10.6, and 5.5%, respectively, after ADTs (Figure S6). While the significant decrease in the ECSA of the Pt/C catalyst is elucidated by particle aggregation or Ostwald ripening (Figure S2) [36], the H-PtCo catalyst exhibits a remarkably stable surface structure even after 10,000 potential cycles due to its well-organized surface layers. Finally, Figure 4d demonstrates that the H-PtCo catalyst exhibits significantly higher mass activities (MAs) at 0.9 V before and after the ADT compared to both Pt/C and C-PtCo catalysts. Furthermore, the reduction rate in the MA of the H-PtCo catalyst during the ADT is the lowest among the catalysts.

As expected, the high stability of the H-PtCo catalyst was clearly confirmed through EDS analysis (Figure S7). For the C-PtCo catalyst, the initial Pt:Co ratio is 83.6:16.4, which shifts to 89.1:10.9 after ADT, indicating a 33.7% reduction in Co content (Figure S7a,b). However, the H-PtCo catalyst exhibits a smaller decrease of 13.3% in Co content (Pt:Co = 75.4:24.6 to 78.7:21.3) during ADT, as shown in Figure S7c,d. These results suggest that the Pt shell on the surface of the H-PtCo catalyst effectively protects the underlying Co from dissolution by acting as a physical barrier, thereby enhancing structural stability.

**Figure 5:**
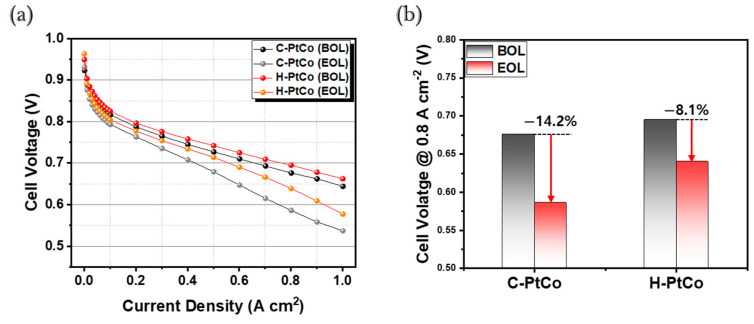
(**a**) Single-cell performance before and after ADTs with 30,000 cycles. (**b**) Change in the voltage of the tested catalysts.

Figure 5a illustrates the single-cell performance test results of the C-PtCo and H-PtCo catalysts employed in the cathode of two MEAs, respectively. As a result, at 0.8 A cm^−2^, the beginning-of-life (BOL) voltage was recorded as 0.675 V for C-PtCo and 0.695 V for H-PtCo, indicating superior fuel cell performance for the H-PtCo catalyst. Additionally, the ADT results after 30,000 cycles reveal a 14.2% decline for the C-PtCo catalyst, while the H-PtCo catalyst exhibits a significantly lower reduction of 8.1% at the end of life (EOL) (Figure 5b). These findings suggest that the H-PtCo catalyst demonstrates superior stability compared to the C-PtCo catalyst, which is consistent with the trends observed in ORR activity from the half-cell tests (Figure 4).

## 4. Conclusions

We proposed a well-engineered H-PtCo alloy catalyst with superior ORR performance, synthesized through a hybrid method in which Co precursors were reduced onto pre-formed colloidal Pt nanoparticles, followed by heat and acid treatments. The Co-enriched sub-surface layer induced strain effects that modulate the electronic structure of the Pt surface, thereby enhancing ORR activity. Additionally, the catalyst surface adopted a Pt-shell configuration, which effectively mitigates transition metal leaching, contributing to its exceptional long-term stability. As a result, the catalyst demonstrated significantly higher mass activity compared to conventional Pt and PtCo catalysts. ADTs revealed a 20.1% performance reduction in the conventional PtCo catalyst, while the H-PtCo catalyst exhibited a more modest decline of 10.4%, indicating superior stability. Single-cell performance evaluations further confirmed that the MEA incorporating the H-PtCo catalyst outperformed that with the conventional PtCo catalyst. Finally, long-term stability tests conducted over 30,000 cycles revealed only an 8.1% voltage degradation for the H-PtCo catalyst, significantly outperforming the conventional PtCo catalyst and demonstrating superior voltage retention. Therefore, we believe that this study will provide valuable insights into the development of high-performance Pt-based alloy nanoparticles for next-generation hydrogen fuel cell systems.

## Data Availability

No data were used for the research described in the article.

## Supplementary Materials

The following supporting information can be downloaded at: https://www.mdpi.com/article/10.3390/nano15090657/s1, Figure S1. Particle size distribution of (a) C-PtCo as-prepared, (b) C-PtCo heat-treated, (c) C-PtCo acid-treated, (d) H-PtCo As-prepared, (e) H-PtCo heat-treated, (f) H-PtCo acid-treated. Figure S2. TEM images and particle size distribution of (a) Pt/C, (b) C-PtCo, and (c) H-PtCo catalysts after ADTs. Figure S3. XRD patterns of (a) as-prepared, (b) heat-treated, and (c) acid-treated C-PtCo and H-PtCo catalysts. The XRD pattern of Pt/C is also included in each figure for comparison. Figure S4. High-resolution Pt 4f XPS spectra for Pt/C, C-PtCo, and H-PtCo. Figure S5. Tafel plots for the ORR of C-PtCo and H-PtCo catalysts. The Tafel slopes were determined to be 83.9 mV dec⁻^1^ for C-PtCo and 75.2 mV dec⁻^1^ for H-PtCo. Figure S6. Cyclic voltammograms (CVs) of (a) C-PtCo, (b) H-PtCo, and (c) commercial Pt/C catalysts before and after ADTs. Figure S7. Comparison of Pt and Co content in C-PtCo and H-PtCo catalysts before and after ADT, based on EDS analysis. (a,b) C-PtCo catalyst before and after ADT, (c,d) H-PtCo catalyst before and after ADT.
