# Supplementary material for: Tailoring Co Distribution in PtCo Alloys for Enhanced Oxygen Reduction Reaction Activity and Durability in Fuel Cells
Source: Nanomaterials (Basel). 2025 Apr 26;15(9):657. doi: 10.3390/nano15090657 (PMC12073838; doi:10.3390/nano15090657)
Supplement: Supplementary file 1 [file nanomaterials-15-00657-s001.zip › nanomaterials-3590327-supplementary.pdf]

Supplementary Information

Tailoring Co Distribution in PtCo Alloys for Enhanced Oxygen Reduction Reaction Activity and Durability in Fuel Cells

Jinhee Lee[1,2], Miso Kim[1], Bongho Lee[1], Jeonghee Jang[1], Suhwan Lee[1], Dae Jong You[1], Juseok Song[1,2], and Namgee Jung[2,*]

[1]Carbon Inc, 4, Techno 2-ro, Yoseong-gu, 34015, Daejeon, Republic of Korea; misokim@thecarbon.studio (M.K.); leebh@thecarbon.studio (B.L.); chemswatt@thecarbon.studio (J.J.); limm741@thecarbon.studio (S.L); dae-jong73@thecarbon.studio (D.J.Y.); juseok@thecarbon.studio (J.S.);
[2]Graduate School of Energy Science and Technology (GEST), Chungnam National University, 99 Daehak-ro, Yuseong-gu, Daejeon, 34134, Republic of Korea; ljh001@thecarbon.studio (J.L.)
*Correspondence: njung@cnu.ac.kr (N.J.)

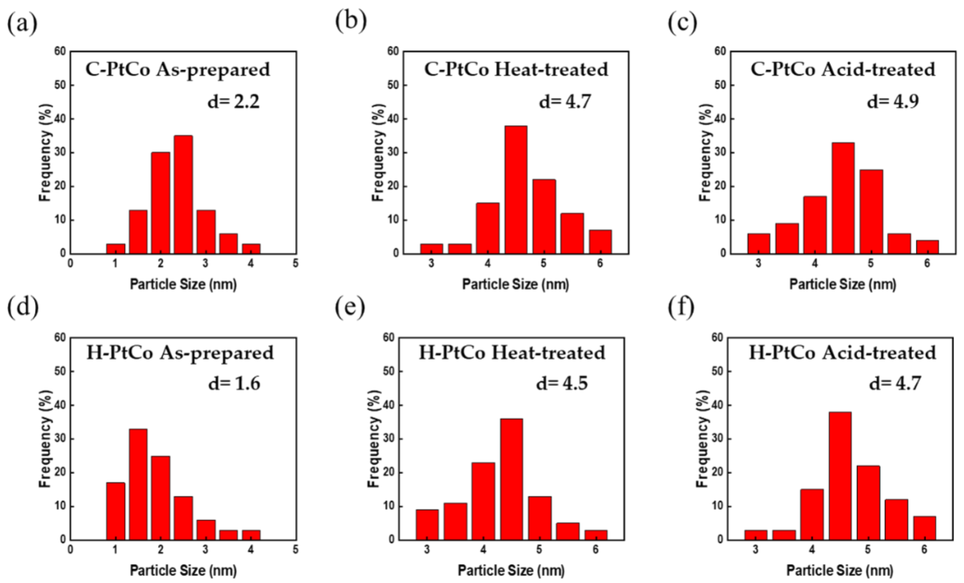

Figure S1. Particle size distribution of (**a**) C-PtCo As-prepared, (**b**) C-PtCo Heat-treated, (**c**) C-PtCo Acid-treated, (**d**) H-PtCo As-prepared, (e) H-PtCo Heat-treated, and (f) H-PtCo Acid-treated.

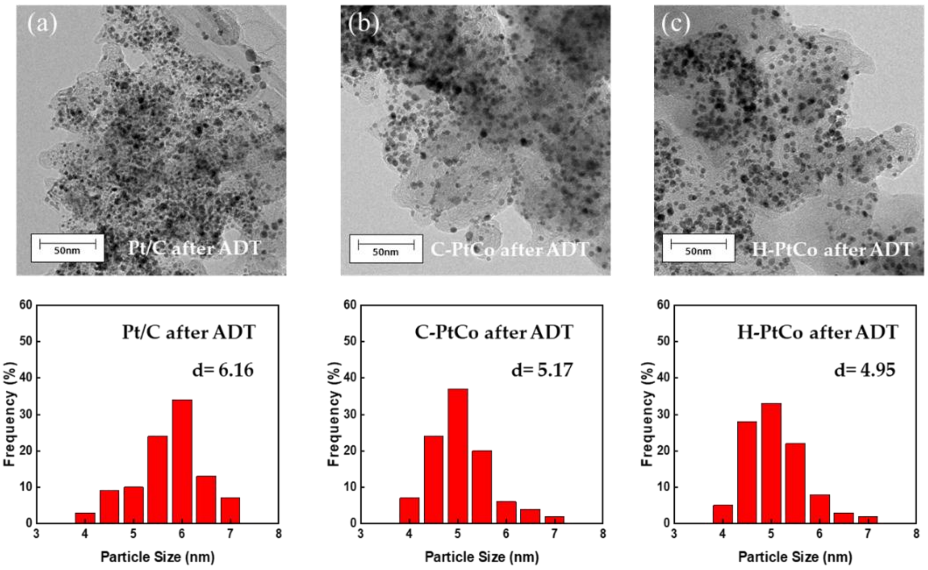

Figure S2. TEM images and particle size distribution of (**a**) Pt/C and (**b**) C-PtCo, and (**c**) H-PtCo catalysts after ADTs.

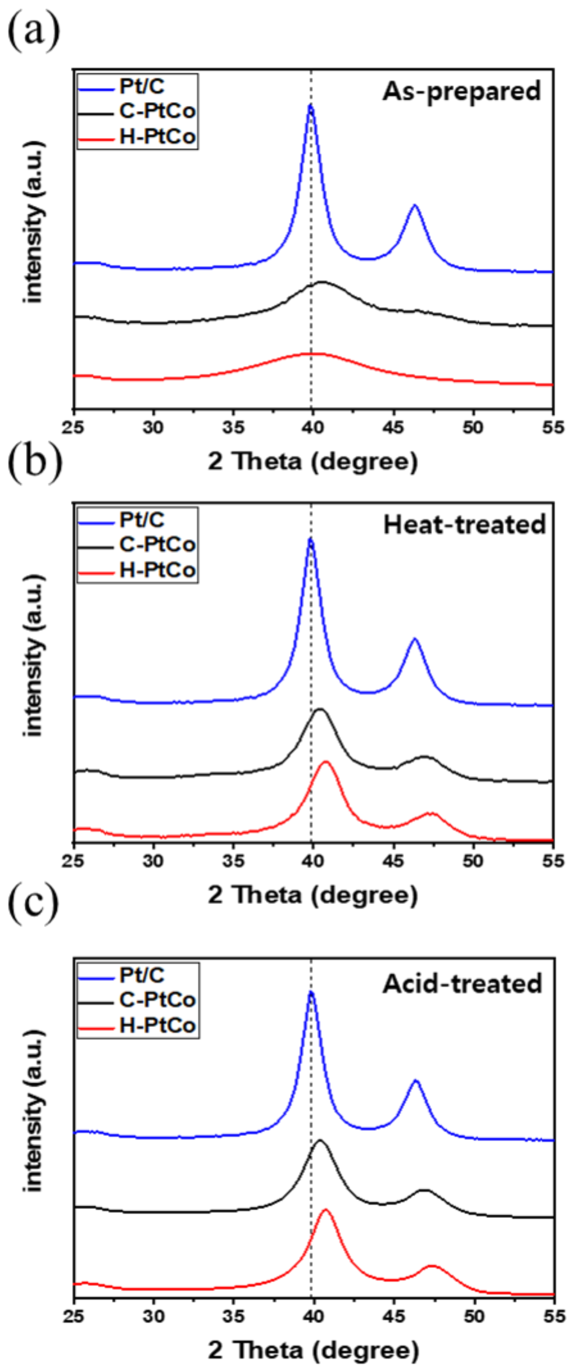

Figure S3. XRD patterns of (a) as-prepared, (b) heat-treated, and (c) acid-treated C-PtCo and H-PtCo catalysts. The XRD pattern of Pt/C is also included in each figure for comparison.

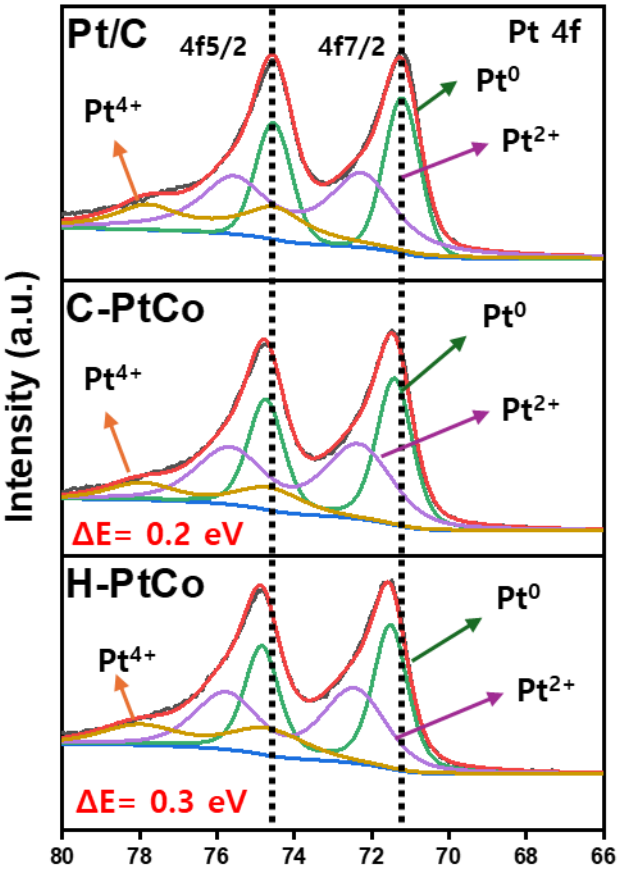

Figure S4. High-resolution Pt 4f XPS spectra for Pt/C, C-PtCo, and H-PtCo.

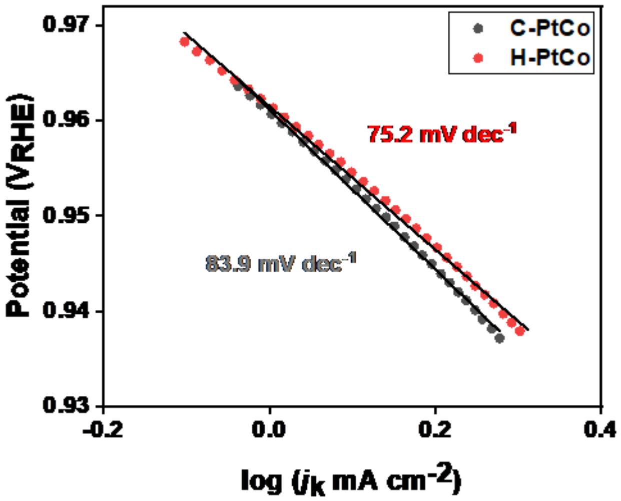

Figure S5. Tafel plots for the ORR of C-PtCo and H-PtCo catalysts. The Tafel slopes were determined to be 83.9 mV dec^{-1} for C-PtCo and 75.2 mV dec^{-1} for H-PtCo.

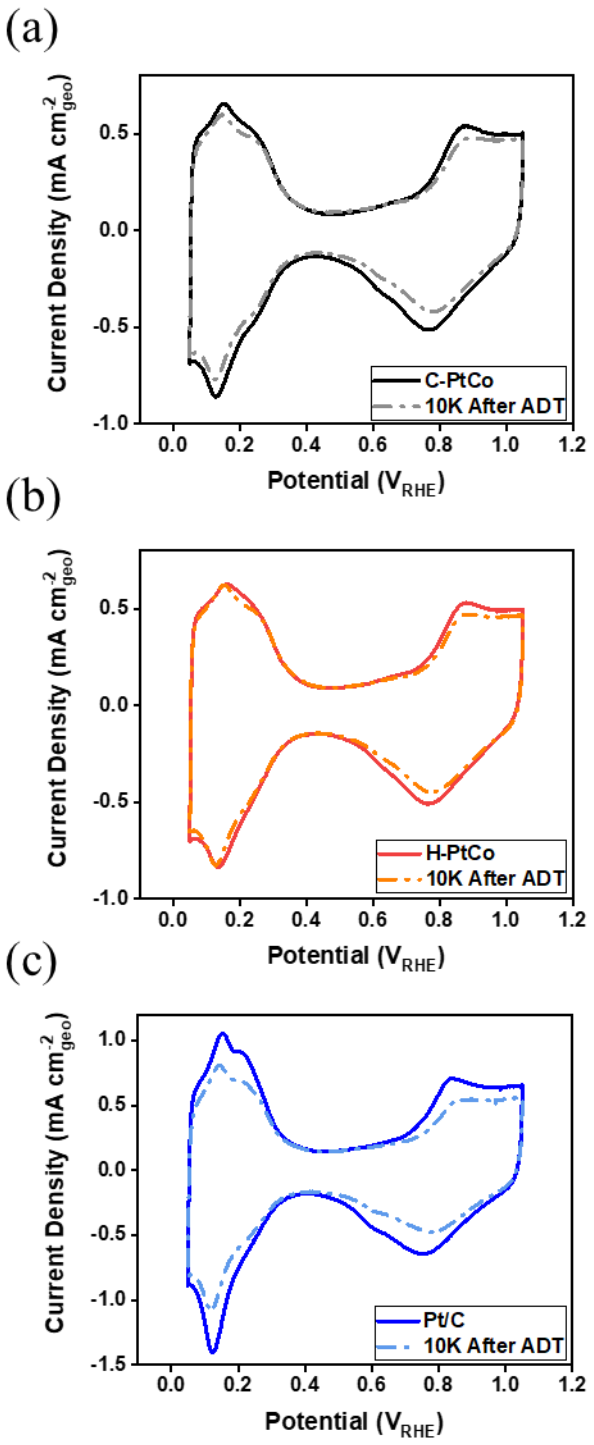

Figure S6. Cyclic voltammograms (CVs) of (a) C-PtCo, (b) H-PtCo, and (c) commercial Pt/C catalysts before and after ADTs.

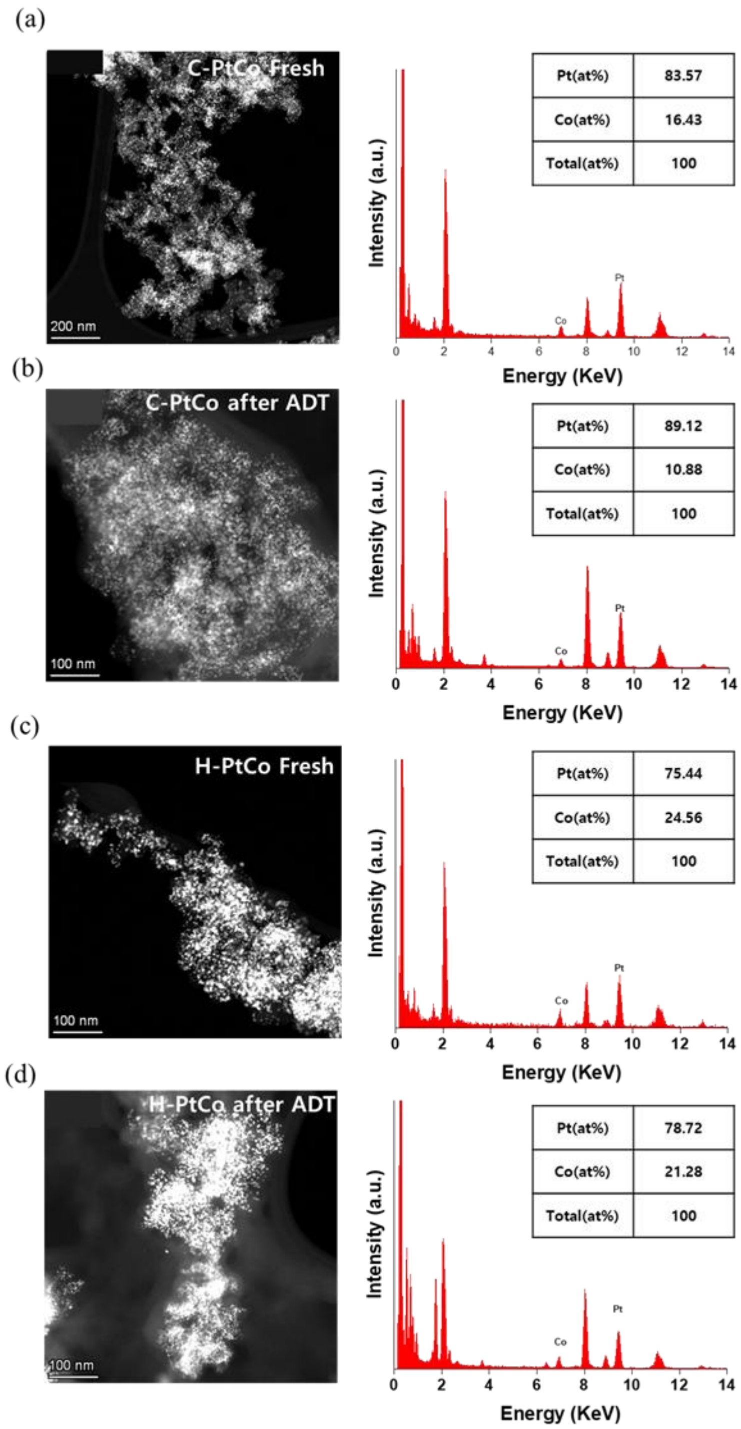

Figure S7. Comparison of Pt and Co content in C-PtCo and H-PtCo catalysts before and after ADT, based on EDS analysis. (a,b) C-PtCo catalyst before and after ADT, (c,d) H-PtCo catalyst before and after ADT.
